# Veratrum Viride in Pulmonary Hemorrhage

**Published:** 1872-09

**Authors:** H. N. Eastman

**Affiliations:** Geneva, N. Y.


ART. III.—*Veratrum Veride in Pulmonary Hemorrhage.* By H. N. Eastman, M. D., Geneva, N. Y.

**Read before the Central New York Medical Association.**

I purpose, in the following paper, to limit my remarks entirely to the hæmostatic effects of veratrum veride in pulmonary hemorrhage, and especially to that form of the disease generally termed passive, as illustrated by the following cases.

In Sept. 1868 Mr. W. C. a theological student, born in England, some twenty-seven years of age, who evidently inherited a strumous constitution from one or both his parents, consulted me in reference to repeated hemorrhages of the lungs, that had occured within the last few days.

The loss of blood up to this time had been trifling, a mere spitting of blood occasionally.

His complexion was rather sallow, his hair dark; he was of small stature, weighing perhaps less than 130 lbs., with a very narrow chest. Percussion elicited a dull sound under the left clavicle, and there was evidently some depression of the ribs in that region. On applying the ear to the part, the respiration was somewhat rough, or at least, did not yield the smooth respiratory number of a normal lung.

On enquiry I learned, that for several years past, the patient had been subject to an occasional slight cough, and very rarely, to trifling hemorrhages, with now and then attacks of indigestion.

He had been strictly moral and temperate in his habits, as far as I could ascertain, or judge from appearances. His occupation had been sedentary, having been employed in teaching or writing for the last several years; more recently he had devoted himself to study.

His pulse, at this time, was not at all accelerated, not exceeding seventy to the minute, soft and compressible; neither was the respiration hurried, to any appreciable extent. I administered a gentle alterative to be taken that night, and followed next morning by saline laxative; and further enjoined perfect quiet, acidulated mucillaginous drinks with a mild astringents and opiates.

Things remained much the same for a day or two, after which, there occurred a profuse hemorrhage without any apparent exciting cause, filling a small bowl half full of florid blood and mucus. These paroxyoms of bleeding persisted in returning every few hours for several days in succession, with unabated violence, notwithstanding every effort to arrest their course and prevent their recurrence, such as plunging the feet and hands in hot stimulating baths, administering, in succession, free doses of Gallic acid, Acetate of lead and Opium, per Sulphate of Iron, Sulphate of Zinc, and, indeed almost every form of mineral and vegetable astringent. No depleting measures or arterial sedatives were, as yet, employed, for the reason that not the least excitement of the circulation was at any time manifest, before or during the bleeding, nor any increased heat of the surface.

No premonitory symptoms foreshadowed a return of the hemorrhage, the pulse before and at the time, remaining perfectly normal in force and frequency, gave no intimation of an increased action of the heart on the one hand, nor any considerable decrease of power on the other, even after repeated profuse hemorrhages, as one would naturally expect, after so large and frequent discharges of blood from the lungs.

Nor was the effect of permanent counter-irritation to the extremities and thorax any more efficacious, neither did large opiates at all control the flow of blood, but the case continued to progress downward, in spite of all the means ordinarily made use of with success, towards a fatal and speedy termination.

In this discouraging state of things, I called in my friend, Dr. Geo. N. Dox, who, after carefully examining the case, advised a persevering continuance of the same course, varying the astringents, from time to time, as each one seemed to fail in meeting the indications, hoping that the case would intimately yield to such a course of energetic treatment; but being myself quite impatient, and despairing of further success in the use of all ordinary means of treatment, I suggested to the doctor the propriety of putting the patient on a heroic course of arterial sedatives, not to moderate any abnormal increase of the circulation, for as already stated, there was none present, nor had there been, at any time previous, but rather to bring down the heart's action as far below the natural standard as might be thought practicable and safe, in order to transmit as little blood as was prudent, though the lungs, in a given time, so as to relieve their capillaries as much as possible from distention, till they could recover their tone, and the ruptured vessels could have time to heal, and thus again be able to tolerate safely the normal quantity of blood circulating through them. The doctor readily consented, and I immediately administered six drops of Tilden's fluid ext. of veratrum veride in a little sweetened water, giving orders to repeat the same thing in four drop doses every three hours provided no vomiting or nausea occurred from its use, till the patient should be again visited, meantime ordering mucillaginous drinks, and vegetable nutrient fluids as supports for the time.

Soon after the second dose, no hemorrhage having occured after the first, the pulse began to fall without any nausea coming on. We continued this course without variation for a day or so. When the pulse fell below forty-five, attended with a "queer feeling" about the heat, as the patient expressed it, no bleeding having as yet returned since the first dose.

At this period, not wishing to depress the heart's action further, lest a coagulum should be the result of too slow movement of the blood through the organ, we continued the same dose at longer intervals, some four hours, and in this way, without further lowering the circulation, we held the heart, "in statu quo," continually for two weeks, meantime supporting the strength of the patient by animal broths, and after a little, allowing a cautious return to solid food at long intervals alternating with vegetable and animal fluids, administering after a time, small quantities of wine and iron as a restoration, together with quinine as a vital nerve tonic.

After two weeks freedom from any recurrence of the hemorrhage, I began to allow the circulation to return gradually to its natural standard, cautiously lessening the dose of the veratrum, or increasing the intervals for two weeks more, at the end of which the pulse had regained its ordinary volume and frequency, after which, no further medication was deemed necessary, save, what seemed prudent, to continue for some time longer, a moderate course of iron and quinine to build up the general system and thus guard it against any subsequent assault of a similar character.

After his recovery I advised him to go to Minnesota, as more congenial to his constitution.

He soon followed my advice, went to the village of Fairbault, Minn., where he pursued his theological studies uninterruptedly, was in due time ordained by Bishop Whipple, and is now a successful preacher and Rector of a church in Frontenac, in the same state enjoying, according to our last letters from him, full health.

Another case to which I shall briefly allude was so similar to that just delineated, in its cause, treatment and final result, that it is unnecessary to spend much time in giving its history and particular management. It occurred in the person of Dr. G. C. a very reputable physician, now however, engaged in other pursuits, a man some fifty-three years old at the time, very spare, though tall, weighing some 120 lbs. only, with originally black hair, dark eyes, very narrow chest, of a frail constitution, subject for several years, to turns of coughing, often lasting several weeks, though generally not so severe as to interfere with his ordinaay business habits, unattended, I think, with any hemorrhage previous to the one referred to. His mother is still living in the enjoyment of good health, though far advanced in years. He has a very large number of mature brothers, all living and in sound health; in short, there exist no indications of tuberculosis among his ancestors or in any of the branches of his family.

For the last two or three years he has been subject to occasional and very violent and protracted turns of urinary calculi, or nephretic colic. In the early part of Nov. 1869 he was suddenly attacked with profuse hemorrhage of the lungs, having some days previous, felt a peculiar sensation in a circumscribed portion of the upper part of the left lung.

After employing revulsives, as the stimulating local baths before alluded to in the other case, and some of the more ordinary internal remedies, as opium, astringents &c., the paroxyoms continuing to recur frequently during the first twenty-four hours. Notwithstanding these remedial measures, I immediately commenced with the veratrum, in a six drop dose, of the flui. ext., and proceeded in the selfsame manner I did in the other case, and pursued the course for the same length of time, some four weeks. In this case, as in the former, there was at no time any arterial excitement which seemed to call for lowering treatment; his pulse ranging about sixty-five, and not exceeding seventy per minute at any time, nor was there even present any superficial heat or febrile action.

My object in using the veratrum here, as in the former instance, was to reduce the circulation as far below the natural standard as I deemed prudent, in order to tax the vessels of the lungs as little as possible, or to move as small a quantity of blood through the pulmonary capillaries in a given time as would suffice to carry on the function of aeration, and sustain the vital forces without hazarding the formation of a heart clot. In this case, as in the other, the same "queer feeling" about the heart was recognized, when the pulse fell below forty-five. The same supporting course was pursued in this instance as in the former; and the same gradual letting up of the hearts action was continuously permitted here, after a week or two, until finally at the end of some four weeks, the circulation was allowed to return to its ordinary standard, by withholding entirely the arterial sedative.

This patient convalesced safely as the other, no return of hemorrhage occurring after the system was brought fully under the influence of the veratrum ; nor has there been any recurrence up to the present time.

Following my advice, the Doctor, as soon as was deemed prudent, went to northern Illinois where he spent some months of the ensuing winter, after which he returned in usual health and resumed his ordinary business habits, and since that time he has continued in the enjoyment of his accustomed health and spirits, being occationally as formerly subject to turns of slight coughing, apparently from ordinary bronchial irritation. His present weight is over 150, more than ever before.

Now, there is nothing new or original in resorting to depleting measures or arterial sedatives in pulmonary and other hemorrhages attended with increased vascular excitement; but I am not aware that any one has employed veratrum veride, or any other similar sedative in pulmonary hemorrhage, when no acceleration of the heart's action was present, for the express purpose of reducing the *normal* circulation to the lowest point compatible with the safety of the patient, for the sole object of giving the pulmonary vessels rest, so to speak, to distend them as little as possible with the incoming blood, and thus prevent further rupture of the capillaries of the part, and afford and opportunity for the vascular lesions already existing to be obviated, by temporarily reducing the normal circulation. If others have pursued this course, or if such a plan of treatment has been recommended by any of the periodicals of the day, it has not come to my knowledge.

I have long since had little confidence in the action of astringents proper in hemorrhage of this kind, nor do I place much more reliance on the combined sedative and astringent operation of acetate of lead and opium.

The nitrate of Potash is not sufficiently prompt and reliable in violent cases, while antimony and ipicac are too liable to nauseate before causing sufficient arterial sedation, and digitalis is now, I believe, almost universally regarded as a heart tonic rather than a sedative but in the veratrum veride, we seem to have just the article to hold the heart temporarily, to lay our hand on that organ, as it were, and thus restrain its action while the pulmonary capillaries have time to recover this tone, and the ruptured branches to unite, and that too, without detracting from the vessels an ounce of blood or depressing the vital energy on the one hand, or doing any considerable injury to the digestive apparatus on the other, since it is ordinally unnecessary to produce nausea or intestinal irritation in order to secure the full sedative influence of the veratrum if judiciously administered.

